# Relationship Between In-Hospital Adverse Events and Hospital Performance on 30-Day All-cause Mortality and Readmission for Patients With Heart Failure

**DOI:** 10.1161/CIRCOUTCOMES.122.009573

**Published:** 2023-07-18

**Authors:** Yun Wang, Noel Eldridge, Mark L. Metersky, David Rodrick, Sheila Eckenrode, Jasie Mathew, Deron H. Galusha, Andrea A. Peterson, David Hunt, Sharon-Lise T. Normand, Harlan M. Krumholz

**Affiliations:** 1 Center for Outcomes Research and Evaluation, Yale New Haven Hospital, New Haven, CT (S.E., J.M., H.M.K., Y.W.).; 2 University of Connecticut School of Medicine, Farmington, CT (M.L.M.).; 3 Hartford Healthcare, Trumbull, CT (A.A.P.).; 4 St. Vincent’s Hospital, Bridgeport, CT (A.A.P.).; 5 Section of Cardiovascular Medicine (H.M.K., Y.W.), Department of Internal Medicine, Yale School of Medicine, New Haven, CT.; 6 Section of General Internal Medicine (D.H.G.), Department of Internal Medicine, Yale School of Medicine, New Haven, CT.; 7 Department of Health Policy and Management, Yale School of Public Health, New Haven, CT (H.M.K.).; 8 Department of Health Care Policy, Harvard Medical School, Boston, MA (S.-L.T.N.).; 9 Department of Biostatistics, Harvard T.H. Chan School of Public Health, Boston, MA (S.-L.T.N.).; 10 Agency for Healthcare Research and Quality (D.R., N.E.), both from the United States Department of Health and Human Services, Rockville, MD.; 11 Office of the National Coordinator for Health Information Technology (D.H.), both from the United States Department of Health and Human Services, Rockville, MD.; 12 Defense Health Agency, Falls Church, Virginia (N.E.).

**Keywords:** heart failure, hospitalization, mortality rate, readmission rates

## Abstract

Hospitals with high mortality and readmission rates for patients with heart failure (HF) might also perform poorly in other quality concepts. We sought to evaluate the association between hospital performance on mortality and readmission with hospital performance rates of safety adverse events.

**METHODS:**

This cross-sectional study linked the 2009 to 2019 patient-level adverse events data from the Medicare Patient Safety Monitoring System, a randomly selected medical records-abstracted patient safety database, to the 2005 to 2016 hospital-level HF-specific 30-day all-cause mortality and readmissions data from the United States Centers for Medicare & Medicaid Services. Hospitals were classified to one of 3 performance categories based on their risk-standardized 30-day all-cause mortality and readmission rates: better (both in <25th percentile), worse (both >75th percentile), and average (otherwise). Our main outcome was the occurrence (yes/no) of one or more adverse events during hospitalization. A mixed-effect model was fit to assess the relationship between a patient's risk of having adverse events and hospital performance categories, adjusted for patient and hospital characteristics.

**RESULTS:**

The study included 39 597 patients with HF from 3108 hospitals, of which 252 hospitals (8.1%) and 215 (6.9%) were in the better and worse categories, respectively. The rate of patients with one or more adverse events during a hospitalization was 12.5% (95% CI, 12.1–12.8). Compared with patients admitted to better hospitals, patients admitted to worse hospitals had a higher risk of one or more hospital-acquired adverse events (adjusted risk ratio, 1.24 [95% CI, 1.06–1.44]).

**CONCLUSIONS:**

Patients admitted with HF to hospitals with high 30-day all-cause mortality and readmission rates had a higher risk of in-hospital adverse events. There may be common quality issues among these 3 measure concepts in these hospitals that produce poor performance for patients with HF.

**WHAT IS KNOWN**

- Hospitals vary in their performance on Centers for Medicare & Medicaid Services 30-day mortality and readmission performance measures for patients with heart failure.

**WHAT THE STUDY ADDS**

- This study shows that patients with heart failure admitted to hospitals with higher 30-day all-cause mortality and readmission rates have a relative 24% higher risk of suffering one or more adverse events during their hospitalization. Patients admitted to hospitals that perform poorly on mortality and readmission measures for patients with heart failure have higher risk of hospital adverse events.

Heart failure (HF) is the most common diagnosis for hospitalizations among older patients and accounts for 13% of all deaths in the United States.^1–3^ National patient safety efforts have focused on the avoidance of preventable adverse events, which has shown improvements over time, yet persistent opportunities for improvement remain.^4^

There is marked hospital variation in mortality and readmission rates and some hospitals perform poorly on both metrics. However, there has been little attention given to the relationship of hospital performance on readmissions and the risk of in-hospital adverse events in patients with HF. It is unclear whether patients admitted to hospitals with higher 30-day all-cause mortality and 30-day all-cause readmission rates, publicly reported by the United States Centers for Medicare & Medicaid Services (CMS) for common conditions,^5^ also have a higher risk of developing an in-hospital adverse event. Such a relationship would suggest the possibility of common quality issues in these hospitals. It would also indicate that the combination of CMS publicly reported metrics might convey information about hospital safety performance. Nevertheless, there is no empirical information available at the national level.

We used data from the Medicare Patient Safety Monitoring System (MPSMS), the nation’s largest patient-safety database comprised of hospital medical record-abstracted adverse events among all-payer patients aged ≥18 years, and hospital mortality and readmission data from CMS, to investigate the association between patients’ risks of adverse events and the hospitals’ mortality and readmission rates for patients with HF. The MPSMS data include myocardial infarction, HF, pneumonia, major surgical conditions, and all-other conditions. We have previously reported the association between hospital performance and adverse events for myocardial infarction and pneumonia.^6,7^ For this study, we focused on research questions at the individual and hospital levels: (1) do patients with HF who receive care at hospitals that have high risks of 30-day all-cause mortality and readmission experience a higher risk for hospital adverse events and (2) do hospitals that perform poorly for 30-day all-cause mortality and readmission also have high rates of in-hospital adverse events.

## METHODS

### Study Sample

The Institutional Review Board at Yale University determined that the requirement for informed consent could be waived. Thirty-day all-cause mortality and readmission data for Medicare patients with HF are publicly available from the CMS Hospital Compare website and can be accessed at https://www.cms.gov/medicare/quality-initiatives-patient-assessment-instruments/hospitalqualityinits/hospitalcompare; due to the restriction of data user agreement, the MPSMS data are not publicly available; the American Hospital Association Annual Survey Database is publicly available and can be accessed at https://www.ahadata.com/aha-annual-survey-database; and the methods used for this study are publicly available.

The MPSMS data, at the individual patient level, include 21 common in-hospital adverse event measures (Table S1) jointly developed by federal agencies and private healthcare organizations.^8^ MPSMS medical records were obtained from the CMS Hospital Inpatient Quality Reporting Program, which includes a multi-stage random sample of all-payer patients aged ≥18 years. All-payer data for patients with HF were available from 2009 to 2019. All hospitals, regardless of size, contributed approximately equal numbers of randomly selected records to the MPSMS. Medical record abstraction was conducted at the CMS Clinical Data Abstraction Center. Based on 80 monthly re-abstractions, the agreement between abstraction and re-abstraction ranged from 94% to 99% for data elements used to identify adverse events.^9^

The CMS all-cause 30-day mortality and readmission data provide information on all acute-care hospitals that treat at least 25 Medicare fee-for-service patients aged 65 years or older during a 3-year period. We restricted the CMS data to hospitals with both CMS mortality and readmission measurements and linked them to MPSMS data at the hospital level. For the 2009 to 2012, 2013 to 2016, and 2017 to 2019 MPSMS data, the hospital performance in 30-day all-cause mortality and readmission rates were obtained from July 1, 2005 to June 30, 2008; July 1, 2010 to June 30, 2013; and July 1, 2013 to June 30, 2016 CMS data, respectively. This linkage ensured that information used to characterize hospital mortality and readmission rates was collected in years before admission dates for patients in the MPSMS data.

### Patient and Hospital Characteristics

Patient characteristics were abstracted from medical records, including demographics age, sex, and self-reported race categorized as Black and Other (includes any identified race not included in the aforementioned categories and mixed race), clinical comorbidities (HF, obesity, coronary artery disease, renal disease, cerebrovascular disease, chronic obstructive pulmonary disease, cancer, diabetes), pressure ulcer present at admission, and smoking status. We included the race information in this study for informational purposes only, it was not used for risk-adjustment. Hospital characteristics were obtained from the 2010 to 2017 American Hospital Association Annual Survey Database, including teaching status, Joint Commission certification status, geographic location, ownership, number of beds, and ability to perform coronary artery bypass graft surgery and percutaneous coronary intervention. For hospitals with missing characteristics (n=4), we used additional publicly available data sources, including CMS hospital performance data, to obtain their characteristics. Missing information on the number of beds (0.03%) was imputed using multiple imputation with 10 imputations.

### Hospital Performance

We used the hospital-specific risk-standardized 30-day all-cause mortality and readmission rates as a proxy to represent hospital performance on patient outcomes. CMS estimates these 2 outcomes based on the risk-standardized method for profiling hospitals^10^ (text A in the Supplemental Material). We classified each hospital in the MPSMS into one of 3 mutually exclusive categories by its risk-standardized mortality and readmission rates that were reported in each reporting period: (1) better, if both mortality and readmission rates were <25th percentile of the overall rates; (2) worse, if both rates were >75th percentile of the overall rates; and (3) average, if otherwise.

### Outcome

Our main outcome was the occurrence (yes/no) of one or more adverse events during hospitalization. All patients were at risk for at least 2 adverse events (in-hospital falls and hospital-acquired pressure ulcers) but only varying subsets of patients were at risk for other events (eg, only patients who received warfarin were at risk for a warfarin-associated adverse event).

### Statistical Analysis

We performed descriptive analysis to quantify the differences in patient and hospital characteristics, and the adverse-event rate across hospital performance categories. To evaluate the association between patients’ risks of adverse events and their admitting hospitals’ mortality and readmission rates, we fit a mixed-effect model with a logit-link function and random hospital intercepts to model the occurrence of one or more adverse events during a patient’s hospitalization as a function of the admitting hospital performance, (worse and average) referring to the better category, adjusting for patient characteristics described previously. We converted the odds ratio to a relative risk using the Zhang and Yu method^11^ to represent the difference in relative risk in patients’ adverse events across the hospital performance category.

To address a potential bias resulting from those patients who were admitted to a worse hospital having different baseline characteristics than those who were admitted to other hospitals, we conducted a sensitivity analysis by calculating a stabilized inverse probability weighting, based on the propensity score approach,^12–14^ to estimate the conditional probability of a patient being admitted to a hospital with a given performance category based on their characteristics included in the previous model, plus whether admission occurred on a weekend day, distance from home to hospital, and whether an individual patient was at risk for each of the 21 adverse events. We then refit the mixed-effect model weighted by the stabilized inverse probability weighting.

To address a potential bias resulting from the CMS mortality and readmission rates being calculated solely on Medicare patients aged ≥65, and patients in MPSMS data being ≥18 years, we conducted an additional sensitivity analysis restricting MPSMS data to patients aged ≥65 and repeated the analyses. All models adjusted for hospital characteristics and included an ordinal time variable, ranging from 0 (year, 2009) to 10 (year, 2019) to account for potential secular trends in adverse events. Statistical tests used a 2-sided α of 0.05. No adjustment for multiplicity of testing was made. All analyses were conducted using SAS Version 9.4. The study followed the guidelines for cohort studies, described in the STROBE (Strengthening the Reporting of Observational Studies in Epidemiology) statement: guidelines for reporting observational studies.^15^

## RESULTS

### Study Sample

**Figure 1.**
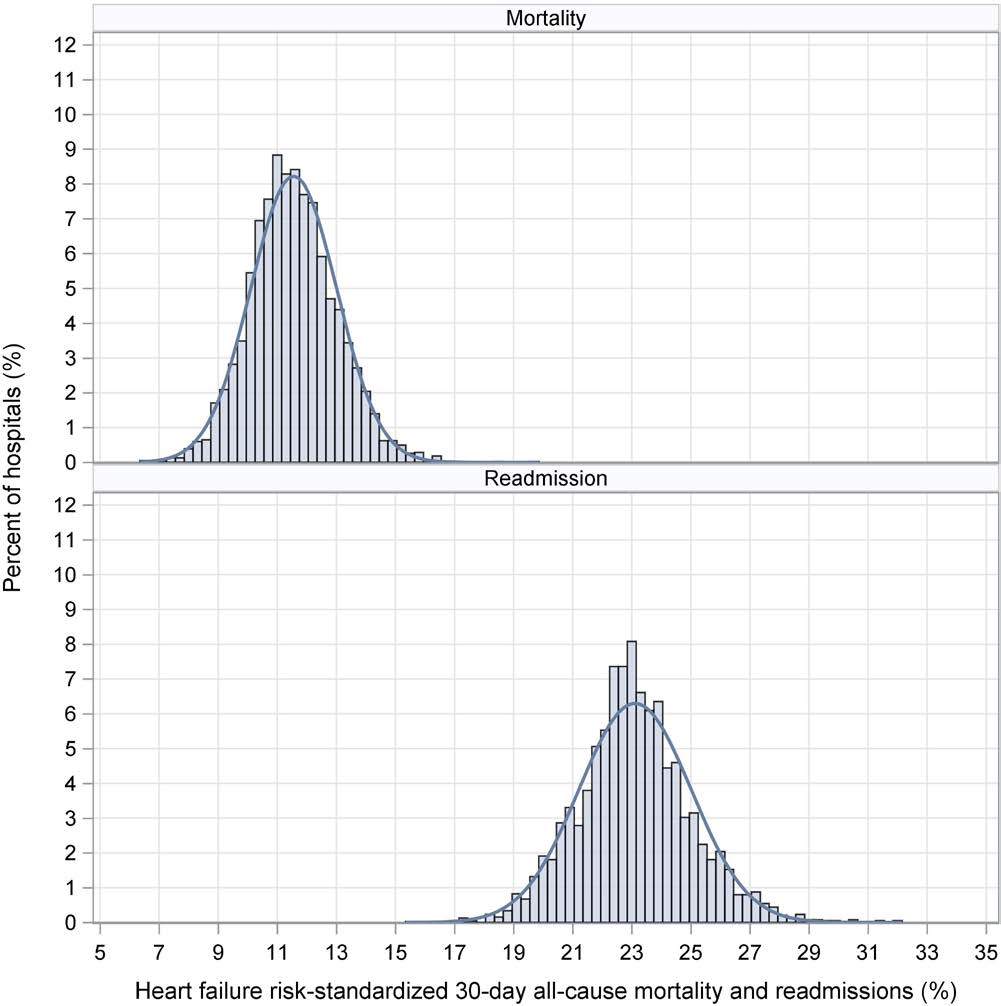
Distributions of hospital-specific risk-standardized 30-day mortality and readmissions.

**Figure 2.**
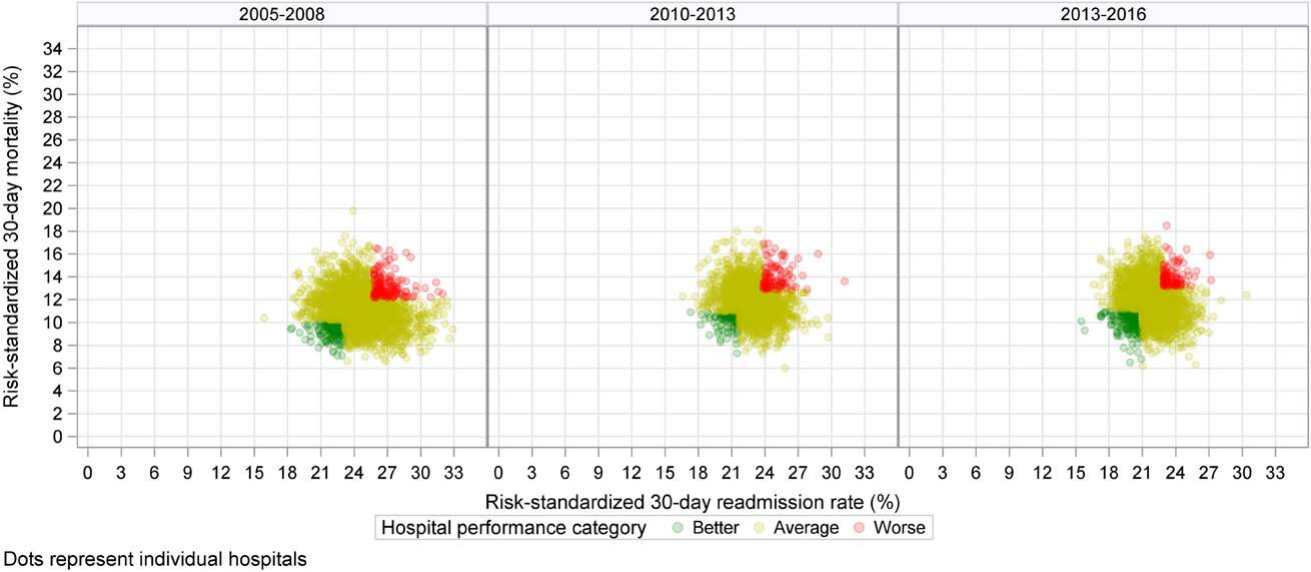
**Distributions of hospital performance categories over time.** Hospital performance was measured by its mortality and readmission rates; better: both mortality and readmission rates <25th percentile of national average; worse: both mortality and readmission rates >75th percentile of national average; and average all others.

**Figure 3.**
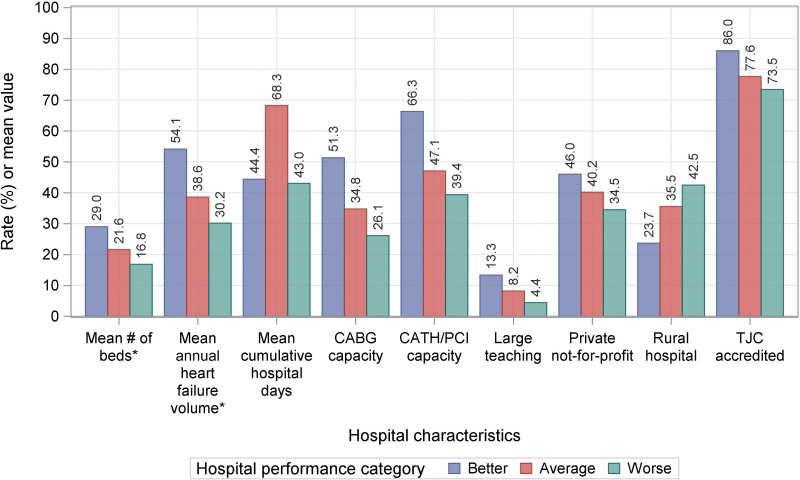
**Hospital characteristics by hospital performance category.** Hospital performance was measured by its mortality and readmission rates; better: both mortality and readmission rates <25th percentile of national average; worse: both mortality and readmission rates >75th percentile of national average; and average all others. *The better group represents 2493 hospitals; The worse group represents 1909 hospitals; and the average group represents 35 195 hospitals. CABG indicates coronary artery bypass graft surgery; CATH, cardiac catheterization; PCI, percutaneous coronary intervention; and TJC, the Joint Commission.

The final sample included 39 597 patients with HF across 3108 acute-care hospitals in the United States. The means (SD) of the hospital-specific risk-standardized 30-day all-cause mortality and readmission rates were 11.4% (1.56) and 23.8% (2.14), respectively (Figure 1). The Pearson Correlation Coefficient between risk-standardized mortality and readmission rates was 0.025 (95% CI, 0.006–0.057). Overall, 252 (8.1%) and 215 (6.9%) hospitals were in the better and worse categories, respectively (Figure 2). Hospitals in the better category were more likely to be large-teaching, private, Joint Commission-certified, not-for-profit, and nonrural, and to have more beds and a high volume of HF hospitalizations (Figure 3).

Among 39 597 patients with HF, 2493, 35 195, and 1909 were in the better, average, and worse categories, respectively. The overall mean (SD) age was 73.5 (14.3) years; 51.1% were female, 77.5% were White patients, 16.7% were Black patients, and 5.8% were other-race (Table 1). These patients were at risk for 277 685 adverse events; each patient was at risk for a mean (min–max) of 6.0 (3–17) adverse events, and the hospital-specific median (interquartile range) number of adverse events for which patients were at risk was 66 (17–120). Patient characteristics were comparable across the 3 hospital categories (Table 1).

**Table 1.**
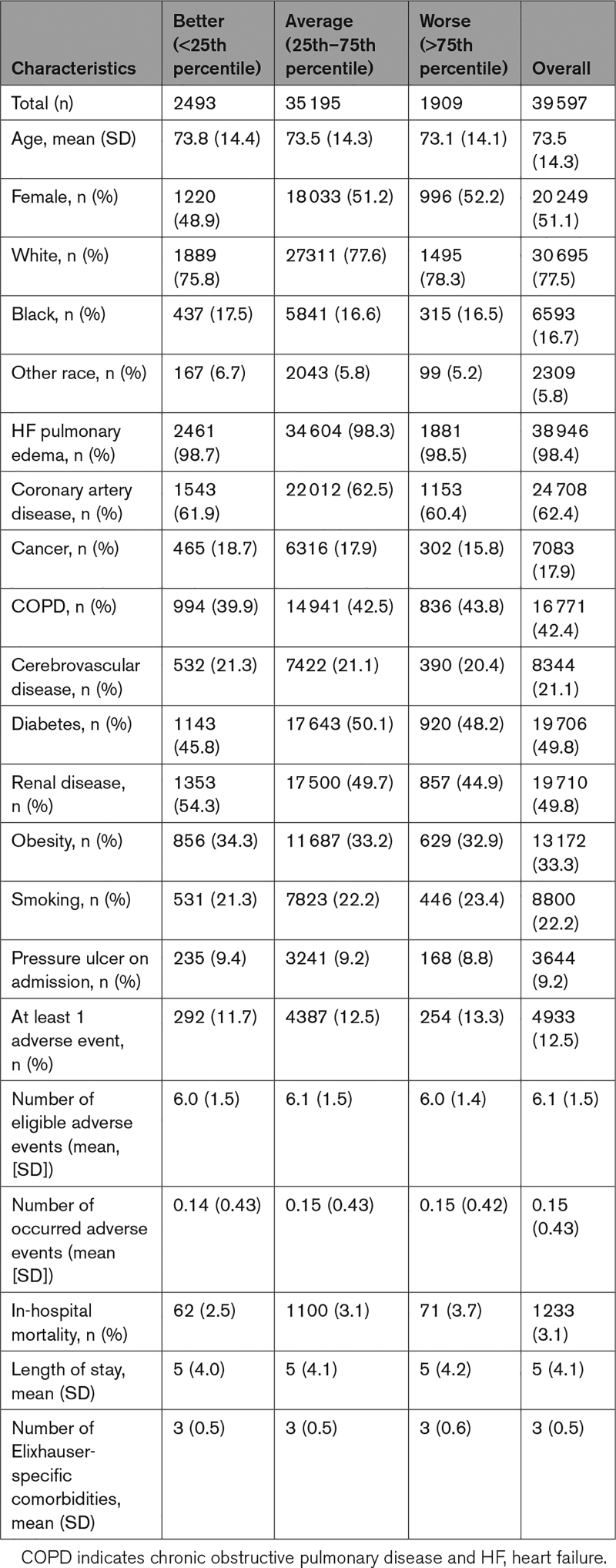
Patient Characteristics by Hospital Performance Category

### Adverse Events and Hospital Performance

**Figure 4.**
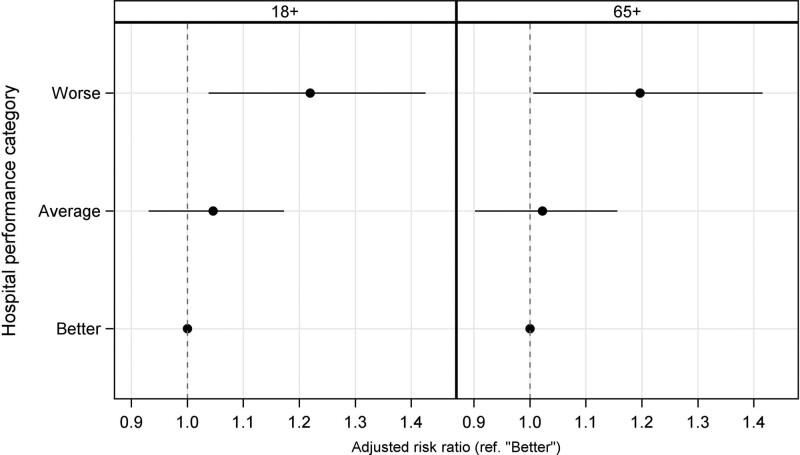
**Association between hospital performance and the risk of developing in-hospital adverse events for patients aged >18 years and aged >65 years.** Hospital performance was measured by mortality and readmission rates; better: both mortality and readmission rates <25th percentile of national average; worse: both mortality and readmission rates >75th percentile of national average; and average all others.

The observed rate of having suffered one or more adverse events during a hospitalization was 12.5% (95% CI, 12.1–12.8). For the better, average, and worse categories, the adverse event rate was 11.7% (95% CI, 10.5–13.3), 12.5% (95% CI, 12.1–12.8), and 13.3% (95% CI, 11.7–14.3), respectively. After accounting for patient and hospital characteristics, patients admitted to a worse hospital had a 23% (adjusted risk ratio, 1.23 [95% CI, 1.04–1.43]) higher risk of adverse events, compared with patients admitted to a better hospital (Figure 4, left panel). There was no difference in the risk of adverse events between patients treated in an average and better hospital (adjusted risk ratio, 1.06 [95% CI, 0.94–1.19]). The inverse probability weighting analysis showed equivalent results. Patients who were admitted to a hospital in the worse category had a 24% (adjusted risk ratio, 1.24 [95% CI, 1.06–1.44]) higher risk of adverse events than patients admitted to a better hospital (Figure S1, left panel). The additional sensitivity analysis, which restricted MPSMS data to patients aged ≥65, included 29 381 patients admitted to 3009 hospitals and showed similar results. Patients admitted to a worse hospital had a relative 20% (adjusted risk ratio, 1.20 [95% CI, 1.01–1.42]) higher risk of adverse events, compared with patients admitted to a better hospital (Figure 4, right panel). The inverse probability weighting analysis also showed that patients who were admitted to a hospital in the worse category had a 21% (adjusted risk ratio, 1.21 [95% CI, 1.01–1.43]) higher risk of adverse events than patients admitted to a better hospital (Figure S1, right panel).

## DISCUSSION

In this study, we found that patients admitted with HF to hospitals that had high rates of 30-day all-cause mortality and readmission had a higher risk of in-hospital adverse events compared with patients admitted with HF to other hospitals. This finding is consistent with the presence of quality issues in these hospitals that affect all 3 metrics—mortality, readmission, and safety-related adverse events. The poor performance on the publicly reported measures conveys information about safety performance.

Our finding is consistent with several possible explanations. The association may be through a common pathway. Such a relationship would be plausible, as quality issues related to mortality and readmission might reasonably also be related to safety. In fact, the 30-day all-cause mortality and readmission risk might be mediated, in part, through safety practices. It is also possible that quality features, such as leadership, communication, and culture, could affect all 3 metrics separately. It may also be that unmeasured factors, such as case mix, that are not accounted for in the models confound the relationship between hospital performance classification and adverse event risk. Because 30-day all-cause mortality and readmission are not strongly related, those with poor performance on both metrics are distinctly different from average. The lack of correlation between mortality and readmission has been attributed to the differences in what may influence mortality from admission in comparison to readmission among those who survived the hospitalization. The fact that hospitals that perform worse on both measures also have higher rates of adverse events is consistent with the hypothesis that the mortality and readmission measures are picking up a signal of overall quality in these hospitals.

Our study, based on medical record-abstracted patient safety information across the country, demonstrated the alignment between hospital performance on 30-day all-cause mortality and readmission and adverse events for patients hospitalized with HF. It provides evidence of room for improvement for hospitals with high rates for both 30-day all-cause mortality and readmissions. The findings extend previous studies^7,16,17^ that focused on the hypothesis that patients with adverse events are at high risk for 30-day all-cause mortality and readmissions. Nevertheless, this study suggests that not only are patients who experience adverse events more likely to experience mortality and readmission, but patients treated at a hospital with high 30-day all-cause mortality and readmission rates may also be at a greater risk of developing adverse events. These outcomes should be a focus of improvement efforts—and it may even be that reducing the adverse events could be a means to improve mortality and readmission rates.^18–20^

Our study has several limitations. We focused on adverse events that were both detected and documented during the index hospitalization and were unable to identify events that may have occurred but were not documented. Restricted by the sample size of the MPSMS data, we were not able to determine if some of the adverse events have stronger relationships with mortality or readmission than others. Adverse events may also be associated with complexity of care at the hospital level; however, our data do not include such information. It is possible that there is a proportion of the adverse events detected in the MPSMS that were not preventable; however, each of these 21 adverse event types is characterized as being frequently preventable. We recognize as well that some adverse events fall outside those defined in the MPSMS. Restricted by lack of an occurrence date for some adverse events, our analysis was conducted based on a logistic model. Nevertheless, as the occurrence date for an adverse event was not the same for all patients, a survival model would be more appropriate for such an analysis. Likewise, we could be missing adverse events for patients who were discharged early. Still, this study distinguishes itself by the breadth and standardization of events measured and its national scope.

In conclusion, patients admitted to a hospital with higher 30-day all-cause mortality and readmission rates have a higher risk of adverse events, strengthening the evidence that 30-day all-cause mortality and readmissions reflect the quality of hospital care. Hospitals that perform poorly on 30-day all-cause mortality and readmission for patients with HF should recognize that they may also have higher risk of hospital adverse events, which would be a target for intervention.

## ARTICLE INFORMATION

### Sources of Funding

This work was supported by contract HHSA290201800005C from the Agency for Healthcare Research and Quality (AHRQ), United States Department of Health and Human Services, Rockville, MD. The authors are solely responsible for this document’s contents, findings, and conclusions, which do not necessarily represent the views of AHRQ. Readers should not interpret any statement in this article as an official position of AHRQ or of the United States Department of Health and Human Services.

### Disclosures

In the past 3 years, Harlan Krumholz received expenses and/or personal fees from UnitedHealth, Element Science, Eyedentifeye, and F-Prime. He is a co-founder of Refactor Health and HugoHealth, and is associated with contracts, through Yale New Haven Hospital, from the Centers for Medicare & Medicaid Services and through Yale University from the Food and Drug Administration, Johnson & Johnson, Google, and Pfizer. Dr Normand, S. Eckenrode, and J. Mathew work under contract with the Centers for Medicare & Medicaid Services to develop and maintain performance measures outside this submitted work. Dr Wang had full access to all the data in the study and takes responsibility for the integrity of the data and the accuracy of the data analysis. He is the founder of Boston Deep Data LLC. Dr Metersky has worked on various quality improvement and patient safety projects with the Centers for Medicare & Medicaid Services and the Agency for Healthcare Research and Quality. His employer has received remuneration for this work. The other authors report no conflicts.

### Supplemental Material

Supplemental Methods

Figure S1

Table S1

## Supplementary Material
