# Supplementary material for: Relationship Between In-Hospital Adverse Events and Hospital Performance on 30-Day All-cause Mortality and Readmission for Patients With Heart Failure
Source: Circ Cardiovasc Qual Outcomes. 2023 Jul 18;16(7):e009573. doi: 10.1161/CIRCOUTCOMES.122.009573 (PMC10351904; doi:10.1161/CIRCOUTCOMES.122.009573)
Supplement: Supplementary file 1 [file hcq-16-e009573-s001.pdf]

SUPPLEMENTAL MATERIAL

Relationship between In-hospital Adverse Events and Hospital Performance on 30-day All-Cause Mortality and Readmission for Patients with Heart Failure

Supplemental Methods

Using a hierarchical generalized linear modeling approach that accounts for patient characteristics and permits hospital-level random intercepts, CMS calculates a risk-standardized ratio, defined as hospital-specific "predicted" deaths divided by hospital-specific "expected" deaths, for each hospital. This ratio, multiplied by the overall national mortality rate, is the risk-standardized mortality rate for an index hospital. A higher-than-expected mortality rate is indicated when the risk-standardized rate is greater than the national rate. The same approach is used to calculate a risk-standardized rate for hospital readmissions.

Figure S1. Association Between Hospital Performance on 30-Day Mortality and Readmissions and In-Hospital Adverse Events based on stabilized inverse probability weighting

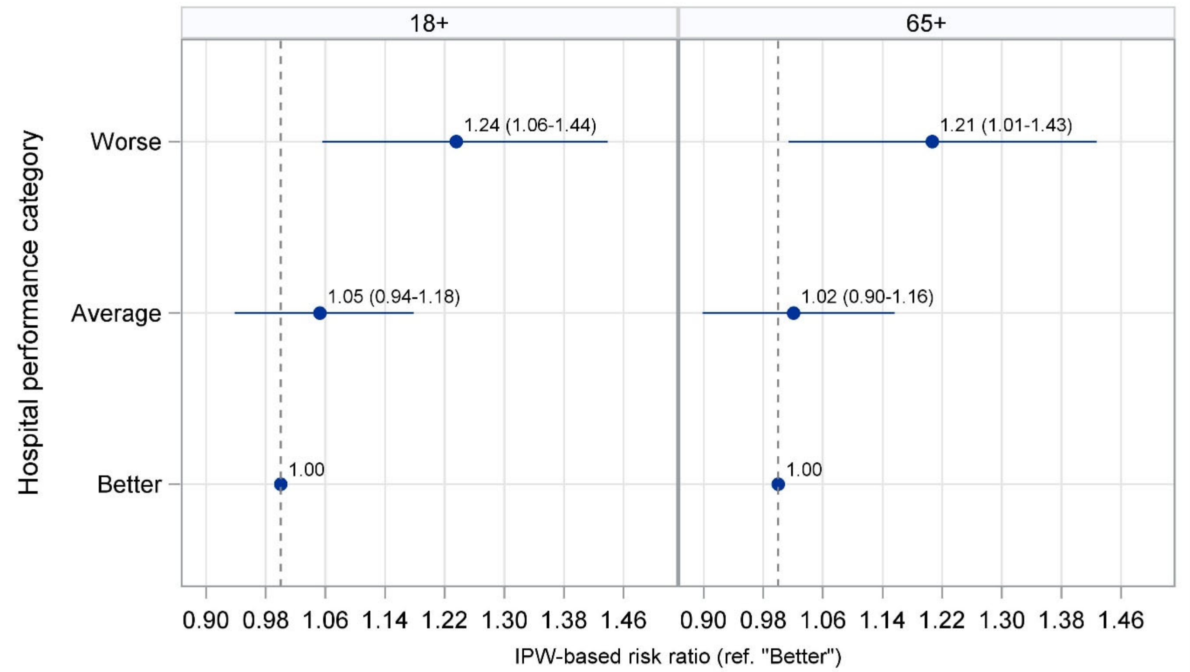

Hospital performance was measured by its mortality and readmission rates
Better: both mortality and readmission rates < 25th percentile of national average, Worse: both mortality and readmission rates > 75th percentile of national average, and Average all others

Table S1. List of the 21 Adverse Event Measures for Which Patients Could Potentially Have Been at Risk During Hospitalizations

Adverse Events Associated with Digoxin
Adverse Events Associated with Hypoglycemic Agents
Adverse Events Associated with Heparin
Adverse Events Associated with Low Molecular Weight Heparin and Factor Xa Inhibitors
Adverse Events Associated with Warfarin
Hospital-Acquired Pressure Ulcers
Inpatient Falls
Central Line-Associated Blood Stream Infections
Postoperative Pneumonia
Hospital-Acquired Antibiotic-Associated Clostridium difficile
Catheter-Associated Urinary Tract Infections
Hospital-Acquired Methicillin-Resistant Staphylococcus aureus
Hospital-Acquired Vancomycin-Resistant Enterococcus
Ventilator-Associated Pneumonia
Adverse Events Associated with Hip Joint Replacement
Adverse Events Associated with Knee Joint Replacement
Mechanical Complications Associated with Central Lines
Postoperative Venous Thromboembolic Events
Postoperative Cardiac Events (Cardiac and Non-cardiac Surgeries)
Adverse Events Associated with Femoral Artery Puncture for Catheter Angiographic Procedures
Contrast Nephropathy Associated with Catheter Angiography
